# Anatomy and Physiology

**Published:** 1818-01

**Authors:** 


### PART III.

### MONTHLY SKETCH of FOREIGN MEDICAL SCIENCE.

——— Sparsa Colligens.

### I. ANATOMY and PHYSIOLOGY.

1. *Malformation.* We have recently had occasion to remark the importance which cases of malformation, rightly viewed and considered, may sometimes acquire from the light thrown by them upon obscure points of the physiology of the animal organs.* It was once a very commonly received opinion, that each ovary, in the female, was exclusively destined to the production of young of a different sex. How such a notion could have originated, we pause not to inquire. We have reason to think that, even in the present day, it is not completely exploded. Of its unsoundness, however, a fact, lately observed, and, doubtless, correctly stated by Professor Chaussier,† constitutes the most direct proof. He examined the body of a woman, who seemed to possess but half of the generative apparatus. One of the ovaries and its corresponding Fallopian tube were nearly imperceptible. Dissection served only to develope some faint rudiments of them buried in, and confounded with, the cellular structure. Yet the woman had borne twelve children of *both sexes*. She had died from diseased heart.

2. *Perforation of the septum ventriculorum* apparently constitutes a rare variety of malformation of the heart: ‡ and an example, published within the last few months by Dr. Delondre, § is the only one with which we are acquainted, wherein the victim of such a defect has nearly attained the adult age. That the aperture of communication, existing in this case, between the ventricles was really a congenital peculiarity, and not a lesion of structure consequent on diseased action, an attentive review of the slight history of it, which our limits only allow us to trace, will, we apprehend, suffice to convince the reader. The subject of the case alluded to, was a lady, aged 19, who had experienced *from her infancy* habitual palpitations of the heart, aggravated by every kind of physical effort and moral affection. From the period of puberty, she sustained attacks of syncope, with vehement palpitation and other formidable symptoms, by which abortion was twice induced, and which progressively increased in violence to the last. The state of the pulse is but once distinctly mentioned: it was then 150, and scarcely perceptible; respiration tumultuous. The ascites and œdema of the limbs, supervening at an advanced stage of the disease, were completely, though not permanently, removed by frictions, with tincture of digitalis employed on the abdomen and inside of the thighs. On dissection, the pleuræ contained four pints, the pericardium about one pint, and the peritonæum (which, in some places, was of a red colour), from six to eight pints of serum. The lungs, particularly the left, were gorged with thick black blood, and had acquired the consistence of liver. The substance of the heart, which exhibited thrice its natural volume, was very flaccid, and readily lacerable. Its right ventricle was extremely thin and much dilated; the left thickened and contracted: and in

---

* See *Medico-Chirurgical Journal and Review*, vol. iv. p. 409.

† *Bulletin de l'Athenée de Médecine de Paris*, Juin, 1817.

‡ *Dr. Farre's Pathological Researches*, Essay i. p. 20.

§ *Observation d'un fait rare de conformation vicieuse du cœur. Recueil periodique de la Société de Médecine de Paris.* Avril, 1817.

the centre of the septum, which divides them, was seen an orifice of communication, measuring at least one inch, of an elliptic figure, and furnished in its whole circumference with bodies of a fibrous structure.

